# Causality of blood metabolites and metabolic pathways on peripheral arteriosclerosis: a Mendelian randomization study

**DOI:** 10.3389/fnut.2024.1421531

**Published:** 2024-09-03

**Authors:** Qian Ye, Yilin Zhou, Kai Xu, Zhili Jiang

**Affiliations:** ^1^Department of Clinical Laboratory, Wenzhou People's Hospital, The Third Affiliated Hospital of Shanghai University, The Third Clinical Institute Affiliated to Wenzhou Medical University, Wenzhou, Zhejiang, China; ^2^College of Engineering, Boston University, Boston, MA, United States; ^3^Cardiac Care Unit, The First Affiliated Hospital of Wenzhou Medical University, Wenzhou, Zhejiang, China

**Keywords:** peripheral arteriosclerosis, Mendelian randomization, blood metabolites, GWAS, causality

## Abstract

**Background:**

Peripheral arteriosclerosis is caused by any atherosclerosis outside the heart and brain. However, the underlying biological mechanisms are not fully understood. This study aims to explore the causal relationship between blood metabolites and peripheral arteriosclerosis.

**Methods:**

A Mendelian randomization (MR) analysis was implemented to estimate the causality of blood metabolites on peripheral arteriosclerosis. A genome-wide association study (GWAS) of 1,400 metabolites was used as the exposure, whereas two different GWAS datasets of peripheral arteriosclerosis were the outcomes. Inverse-variance weighted (IVW) was the main analysis of causal analysis. MR-Egger, the simple mode, weighted median and weighted mode were used to increase the stability and robustness of the results. Cochran Q test, MR-Egger intercept test, the funnel plot, and MR-Pleiotropy RESidual Sum and Outlier were used for sensitivity analyses. Furthermore, metabolic pathway enrichment analysis was performed using MetaboAnalyst5.0.

**Results:**

In this MR study, eight blood metabolites have a strong causal relationship with peripheral arteriosclerosis, including 1-myristoyl-2-arachidonoyl-GPC (14:0/20:4), 1-palmitoyl-2-arachidonoyl-gpc (16:0/20:4n6), 1-(1-enyl-stearoyl)-2-arachidonoyl-GPE, 1-palmitoyl-2-dihomo-linolenoyl-GPC, Gamma-glutamylleucine, Deoxycholic acid glucuronide and two named X- (X-24546, X-26111). In addition, five important metabolic pathways in peripheral arteriosclerosis were identified through metabolic pathway analysis.

**Conclusion:**

This study provides evidence for the causal relationship between blood metabolites and peripheral arteriosclerosis, and these eight blood metabolites provide new perspectives for screening and prevention of peripheral arteriosclerosis in the future.

## Background

Atherosclerosis, which is formed by fibrofatty lesions in the artery walls, is a systemic, progressive inflammatory disease (1). It remains a leading cause of death and morbidity worldwide. When it affects peripheral arteries, it will lead to atherosclerotic renal artery stenosis, multivessel atherosclerotic disease of mesentery, and lower extremity atherosclerosis (2, 3). In recent studies, the degree of systemic inflammation and risk of cardiovascular events in peripheral atherosclerosis are higher than those in coronary atherosclerosis, and the long-term prognosis is poor (4, 5). Age, dyslipidemia, obesity, smoking, high blood pressure and diabetes are considered risk factors for peripheral atherosclerosis (6). However, the prevention measures for peripheral atherosclerosis are still insufficient. Currently, the screening of peripheral atherosclerosis is primarily conducted using ultrasound. However, the accuracy of the results can vary significantly depending on the proficiency of the sonographer. While lower extremity arterial CTA provides more direct and clear diagnostic results, it imposes stricter requirements on the patient’s renal function. Therefore, there is a need for more straightforward indicators to aid in the screening of patients who require intervention.

In recent years, metabolomics has made active contributions to the study of the disease mechanisms. Metabolomics can elucidate biological mechanisms by identifying altered metabolites and metabolic pathways (7). Although several metabolites have been observed to be associated with peripheral arteriosclerosis in population-based cohorts, systematic studies of the causal relationship between blood metabolites and peripheral arteriosclerosis are still unclear (8). In addition, traditional observational studies face challenges in establishing causal relationships due to potential confounding factors.

Recently, Mendelian randomization (MR) analysis is proposed as a new epidemiological tool for studying the causal relationship between disease exposure and clinical outcomes (9). MR uses single nucleotide polymorphisms (SNPs) as an instrumental variable (IV). Also, MR relies on natural random classification of genetic variations during meiosis, resulting in a random distribution of genetic variations. Due to the drawbacks of high cost, long duration, and low feasibility of randomized controlled trials (RCTs), MR can be used as an alternative method to RCTs (10).

Our study aimed to use an MR analysis to fully investigate the causal effect of 1,400 blood metabolites on peripheral arteriosclerosis with pooled data from a genome-wide association study (GWAS).

## Methods

### Study design

**Figure 1:**
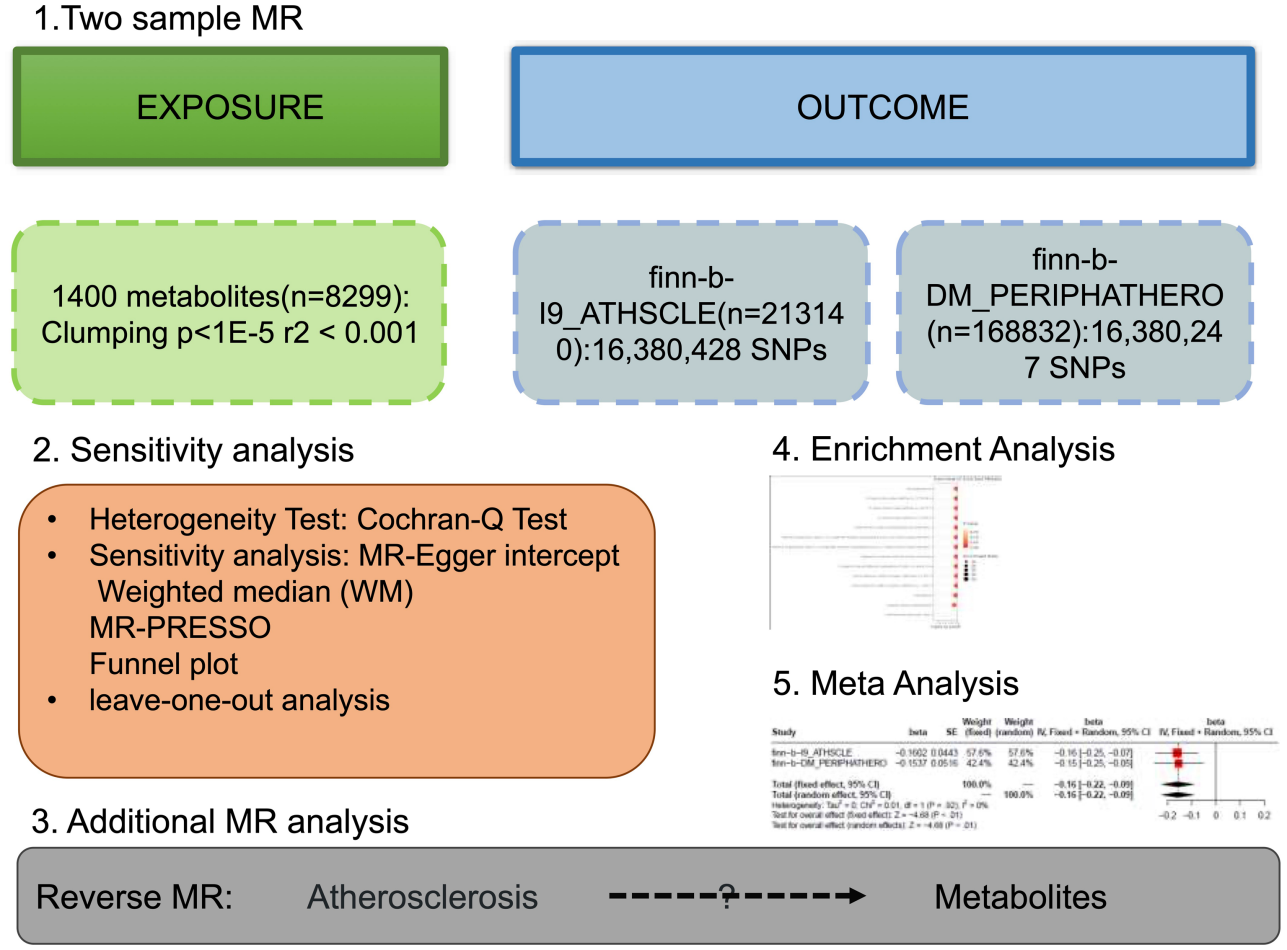
The study design of the study. Detailed information can be found in the main text. MR, Mendelian randomization; SNP, single nucleotide polymorphism.

An effective MR study should meet three assumptions: (1) IVs are highly correlated with interest exposure; (2) IVs are not affected by confounding factors; (3) IVs are not related to results and only affects the outcome through exposure (11). All MR Analyses were carried out using Two Sample MR in R software (4.2.1). The flowchart of this study was shown in Figure 1. STROBE-MR checklist was completed to ensure the integrity of MR Studies (Supplementary Table S1).

### GWAS data for 1,400 blood metabolites and peripheral arteriosclerosis

The genetic data of blood metabolites came from the metabonomics GWAS catalog server.^1^ The report was based on Chen et al.’s genome-wide association scanning with high-throughput metabolic analysis, which ultimately identified nearly 15.4 million SNPs of 1,400 metabolites associated with human genetic variation (12, 13). This study included 8,299 unrelated European subjects from the Canadian Longitudinal Study of Aging (CLSA) cohort. The levels of metabolites from plasma samples were quantified using the Ultrahigh Performance Liquid Chromatography-Tandem Mass Spectroscopy (UPLC-MS/MS) platform. After quality control and batch normalization, the quantification of metabolites was used in further research. The detailed names of 1,400 metabolites were shown in Supplementary Table S2, of which 220 were named X-because their chemical properties were not well defined.

GWAS summary data for peripheral arteriosclerosis were obtained from IEU open GWAS project^2^ with 16,380,428 SNPs which registration number was the finn-b-I9_ATHSCLE, containing 213,140 participants (6,599 cases and 206,541 controls). The finn-b-I9_ATHSCLE dataset, published in 2021, consists of data from the Finnish population. This dataset primarily represents a cohort excluding individuals with lower extremity and cardiovascular atherosclerosis. The replication cohort was also from IEU open GWAS project, with 16,380,247 SNPs. The registration number of the replication cohort was the finn-b-DM_PERIPHATHERO, containing 168,832 participants (6,631 cases and 162,201 controls). The finn-b-DM_PERIPHATHERO dataset, published in 2021 and focuses on a cohort with lower limb atherosclerosis. The above GWAS data for peripheral arteriosclerosis were used for preliminary analysis.

### IVs selection

A series of standards were developed to screen for IV related to blood metabolites. Firstly, SNPs were extracted with significance threshold *p* < 1 × 10^−5^. Then, identification of independent variants was used R software clustering program. We clumped SNPs with clump_kb set to 10,000 and clump_r2 set to 0.001. Finally, the recommended threshold for further MR analysis was *F* > 10.

### Statistical analysis and sensitivity analysis

The causal associations between blood metabolites and arteriosclerosis were mainly evaluated using inverse variance weighting (IVW) method by combining all Wald ratios of IVs (14). Therefore, we initially used IVW-based estimates (*p* < 0.05) to screen for blood metabolites that are causally associated with atherosclerosis. Meanwhile, four methods, including MR-Egger, the simple mode (SM), weighted median (WM) and weighted mode (WMO) were also used to evaluate MR estimates of potential blood metabolites. MR-Egger can detect violations of IVs assumptions, and provide estimates of effects that are not affected by these violations (15). WM is used to combine data from multiple genetic variants into a single causal estimator and gives an accurate estimate based on the assumption that at least 50% of IVs are valid (16). SM provides robustness for pleiotropy and WMO is sensitive to the difficult bandwidth selection for mode estimation (17, 18). At least three MR methods had consistent directions and magnitude, these metabolites were considered candidate metabolite markers.

Sevel sensitivity analyses were performed to assess any deviations from the MR assumptions. Cochran Q test, MR-Egger intercept test, the funnel plot, and MR-Pleiotropy RESidual Sum and Outlier (MR-PRESSO) were used to detect and correct heterogeneity and pleiotropy (15, 19). Cochran Q test-derived *p* < 0.05 was considered to be the heterogeneity of the results (19). MR-Egger was used to identify potential pleiotropy and to assess the impact of pleiotropy on intercept test risk estimates (15). There considered no pleiotropy when *p* > 0.05. MR - PRESSO was used to check again for the presence of heterogeneous SNPs (20). We used the run_mr_presso() function from the TwoSampleMR R package. The results are evaluated based on two criteria: there should be no outliers detected, and the Global Test *p* - value must be greater than 0.05. If these criteria are met, it indicates the absence of horizontal pleiotropy. Leave-one-out (LOO) analysis was performed to detect whether the MR estimates were influenced by individual SNPs.

### Replication and meta-analysis

To comprehensively assess the robustness of candidate blood metabolites, we replicated the IVW analysis in another independent arteriosclerosis cohort, which registration number was the finn-b-DM_PERIPHATHERO. The replication analysis was using the same methods and sensitivity applied in primary analysis. Then, a meta-analysis of two MR analyses with registration number finn-b-I9_ATHSCLE and finn-b-DM_PERIPHATHERO was conducted to determine the final candidates. Meta-analysis is achieved by metafor R package.

### Reverse MR

The following possible scenarios can be used to explain the genetically predicted association between the two phenotypes: (1) a valid causal relationship from phenotype 1 to phenotype 2; (2) a reverse causal relationship from phenotype 2 to phenotype 1; (3) The two phenotypes might share a common genetic structure; (4) There is a linkage disequilibrium (LD) between the major SNPs of the two phenotypes. We performed reverse MR analysis to rule out scenario 2 to assess the causal effect of the arteriosclerosis phenotype on candidate metabolites.

### Enrichment analysis

The corresponding ids of potential metabolites were searched and identified in the Human Metabolome Database. To explore the potential metabolite groups or pathways that may be associated with the biological processes of arteriosclerosis, metabolic pathway enrichment analysis was conducted using MetaboAnalyst5.0’s pathway enrichment function modules^3^ and the *p*-value is not processed.

### Ethical statement

The study used publicly available data. Each study in the GWAS is approved by the appropriate institutional review board, with informed consent provided by the participants or their authorized representatives.

## Results

### Preliminary analysis

**Figure 2:**
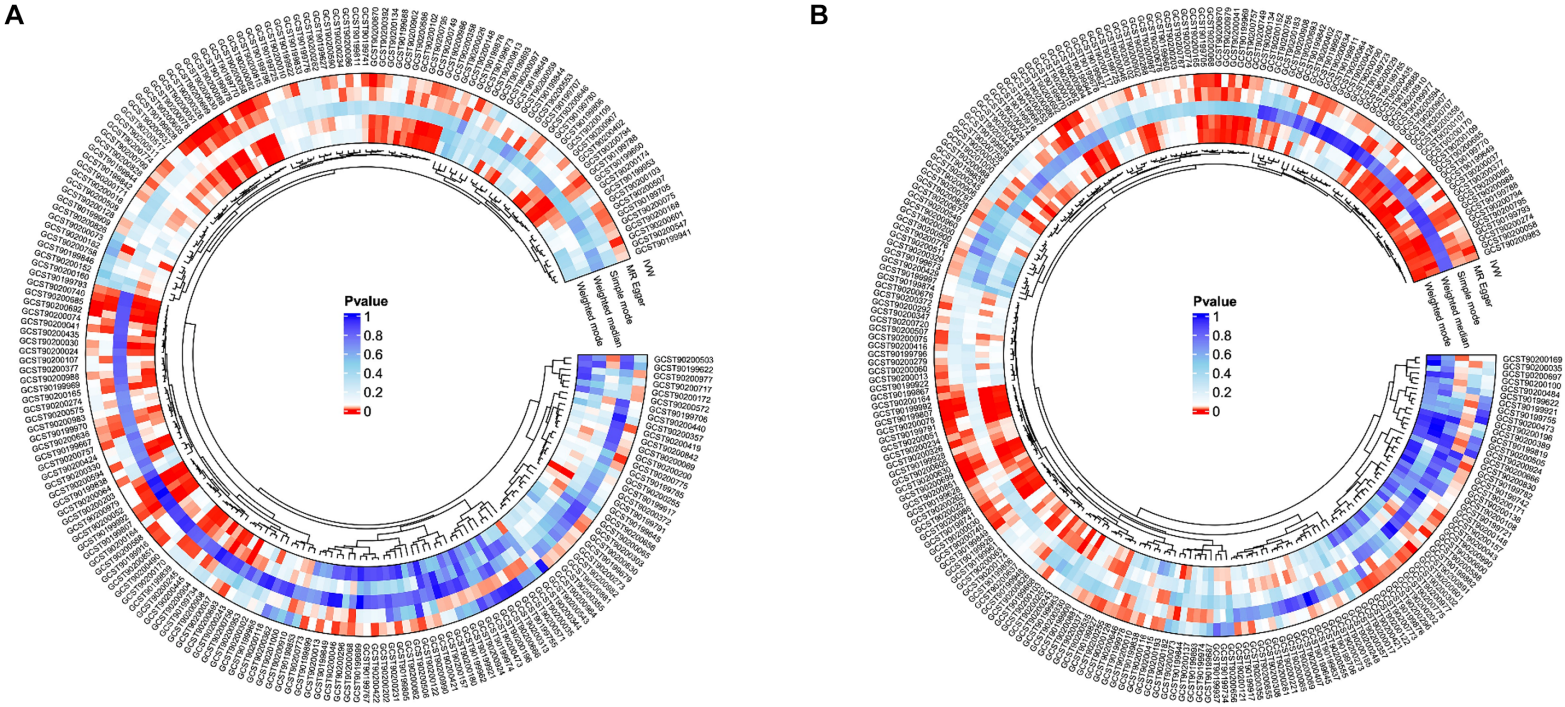
Potential causal metabolites (IVW: *p* < 0.05) for peripheral arteriosclerosis in the GWAS data. **(A)** finn-b-I9_ATHSCLE; **(B)** finn-b-DM_PERIPHATHERO; IVW, inverse variance weighting.

**Figure 3:**
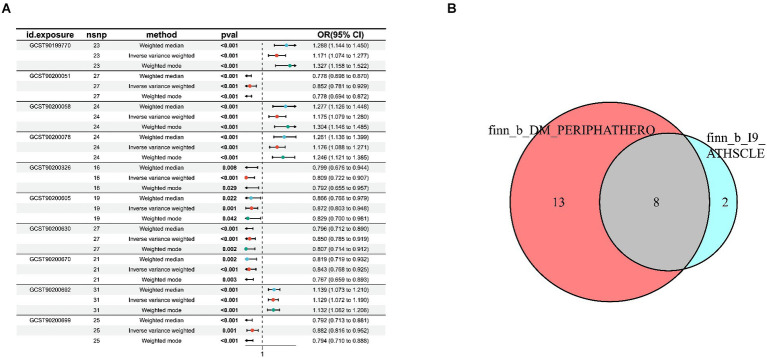
Sensitivity analysis for candidate blood metabolites on peripheral atherosclerosis GWAS databases. **(A)** finn-b-I9_ATHSCLE; **(B)** combined analysis of finn-b-I9_ATHSCLE database and finn-b-DM_PERIPHATHERO database.

In the GWAS data which the registration number was finn-b-I9_ATHSCLE, 233 metabolites were preliminarily identified (Figure 2A). The criteria to narrow down metabolites are as follows: (1) The directionality must be consistent across all Mendelian randomization methods tested. (2) The *p* - values for both MR - PRESSO and pleiotropy must be greater than 0.05. (3) The IVW *p* - value must be less than 0.05. After sensitivity analysis, only 10 of them met the criteria for candidate blood metabolites involved in the development of peripheral atherosclerosis, including 1-arachidonylglycerol (20:4) (OR = 1.171, 95% confidence interval (CI) = 1.074–1.277, *p* < 0.001), 1-palmitoyl-2-dihomo-linolenoyl-GPC (16:0/20:3n3 or 6) (OR = 0.852, 95% CI = 0.781–0.929, *p* < 0.001), 1-(1-enyl-stearoyl)-2-arachidonoyl-GPE (p-18:0/20:4) (OR = 1.175, 95% CI = 1.079–1.280, *p* < 0.001), 1-myristoyl-2-arachidonoyl-GPC (14:0/20:4) (OR = 1.176, 95% CI = 1.088–1.271, *p* < 0.001), Gamma-glutamylleucine (OR = 0.809, 95% CI = 0.722–0.907, *p* < 0.001), X-21845 (OR = 0.872, 95% CI = 0.803–0.948, *p* = 0.001), X-24546 (OR = 0.850, 95% CI = 0.785–0.919, *p* < 0.001), X-26111 (OR = 0.843, 95% CI = 0.768–0.925, *p* < 0.001), 1-palmitoyl-2-arachidonoyl-gpc (16:0/20:4n6) (OR = 1.129, 95% CI = 1.072–1.190, *p* < 0.001), Deoxycholic acid glucuronide (OR = 0.882, 95% CI = 0.816–0.952, *p* = 0.001) (Table 1). In summary, the estimates derived from the IVW were significant (*p* < 0.05), while the IVW, WM and WMO estimates showed consistent direction and magnitude, which supported the robustness of causality (Figure 3A). With the results of negative heterogeneous SNPs in MR - PRESSO, our results suggested that no outliers and pleiotropy exist in the previous analysis, indicating that the metabolite used in the next step of research will not cause bias in further analysis and is more robust. MR - PRESSO results suggested there were no heterogeneous SNPs. Besides, the intercept term of MR-Egger shows a lower risk of horizontal pleiotropy and no heterogeneity was detected by Cochran Q test. Furthermore, the LOO analysis results indicated that the results were not affected by a single SNP.

**Table 1:** MR from blood metabolites on finn-b-I9_ATHSCLE.

	Metabolites	Method	nsnp	se	*p* value	or	or_lci95	or_uci95
GCST90199770	1-arachidonylglycerol (20:4)	MR Egger	23	0.084024	0.006921	1.286007	1.090738	1.516233
		Weighted median	23	0.061672	4.17E-05	1.287508	1.140915	1.452936
		Inverse variance weighted	23	0.044275	0.000362	1.171038	1.073701	1.277198
		Simple mode	23	0.151838	0.161341	1.246163	0.925396	1.678116
		Weighted mode	23	0.065238	0.000262	1.327459	1.168126	1.508526
GCST90200051	1-palmitoyl-2-dihomo-linolenoyl-GPC (16:0/20:3n3 or 6)	MR Egger	27	0.08862	0.026155	0.810987	0.681677	0.964826
		Weighted median	27	0.057025	1.08E-05	0.778062	0.695782	0.870072
		Inverse variance weighted	27	0.044308	0.0003	0.851979	0.781112	0.929276
		Simple mode	27	0.135934	0.054231	0.760288	0.582463	0.992402
		Weighted mode	27	0.058922	0.00024	0.778247	0.693364	0.87352
GCST90200058	1-(1-enyl-stearoyl)-2-arachidonoyl-GPE (p-18:0/20:4)	MR Egger	24	0.08739	0.021217	1.242143	1.046607	1.474212
		Weighted median	24	0.065422	0.000189	1.276621	1.122985	1.451276
		Inverse variance weighted	24	0.043657	0.000222	1.174906	1.078554	1.279866
		Simple mode	24	0.14847	0.116829	1.273706	0.952115	1.703919
		Weighted mode	24	0.064393	0.000414	1.304117	1.149487	1.479548
GCST90200078	1-myristoyl-2-arachidonoyl-GPC (14:0/20:4)	MR Egger	24	0.06793	0.00403	1.243706	1.088666	1.420826
		Weighted median	24	0.051724	7.48E-06	1.260737	1.13919	1.395253
		Inverse variance weighted	24	0.039804	4.66E-05	1.175959	1.087703	1.271377
		Simple mode	24	0.104346	0.046063	1.246125	1.015642	1.528912
		Weighted mode	24	0.048758	0.000157	1.246125	1.132552	1.371087
GCST90200326	Gamma-glutamylleucine	MR Egger	16	0.098258	0.005343	0.723615	0.596855	0.877296
		Weighted median	16	0.088217	0.010967	0.798994	0.672128	0.949807
		Inverse variance weighted	16	0.058102	0.000265	0.809041	0.721961	0.906625
		Simple mode	16	0.124879	0.09198	0.798677	0.625276	1.020164
		Weighted mode	16	0.096295	0.028653	0.792091	0.655854	0.956627
GCST90200605	X-21845	MR Egger	19	0.087899	0.111119	0.862669	0.726144	1.024862
		Weighted median	19	0.061224	0.01852	0.865727	0.767831	0.976103
		Inverse variance weighted	19	0.042297	0.001242	0.872339	0.802937	0.94774
		Simple mode	19	0.103668	0.075716	0.822517	0.671276	1.007832
		Weighted mode	19	0.078633	0.028001	0.828677	0.710316	0.966761
GCST90200630	X-24546	MR Egger	27	0.071122	0.01286	0.826499	0.718956	0.95013
		Weighted median	27	0.055523	4.04E-05	0.796179	0.714082	0.887714
		Inverse variance weighted	27	0.040099	4.82E-05	0.849618	0.7854	0.919087
		Simple mode	27	0.123601	0.094478	0.806929	0.633322	1.028127
		Weighted mode	27	0.055424	0.000655	0.806929	0.723865	0.899526
GCST90200670	X-26111	MR Egger	21	0.092316	0.014167	0.779328	0.650338	0.933903
		Weighted median	21	0.06938	0.003953	0.818775	0.714672	0.938042
		Inverse variance weighted	21	0.047353	0.000309	0.842977	0.76826	0.924961
		Simple mode	21	0.14521	0.261165	0.845427	0.63602	1.12378
		Weighted mode	21	0.076174	0.002375	0.767274	0.66086	0.890823
GCST90200692	1-palmitoyl-2-arachidonoyl-gpc (16:0/20:4n6)	MR Egger	31	0.037183	0.038158	1.08412	1.007921	1.166079
		Weighted median	31	0.029219	8.00E-06	1.139358	1.075941	1.206513
		Inverse variance weighted	31	0.026509	4.41E-06	1.12942	1.072237	1.189652
		Simple mode	31	0.098711	0.844183	1.019763	0.840379	1.237437
		Weighted mode	31	0.028201	0.000131	1.131691	1.070835	1.196006
GCST90200699	Deoxycholic acid glucuronide	MR Egger	25	0.074092	0.003333	0.784605	0.678551	0.907235
		Weighted median	25	0.053065	1.16E-05	0.792376	0.714105	0.879227
		Inverse variance weighted	25	0.039279	0.001368	0.881841	0.816498	0.952413
		Simple mode	25	0.116054	0.080946	0.809412	0.644737	1.016148
		Weighted mode	25	0.063245	0.001283	0.79408	0.701503	0.898876

### Replication and meta-analysis

**Figure 4:**
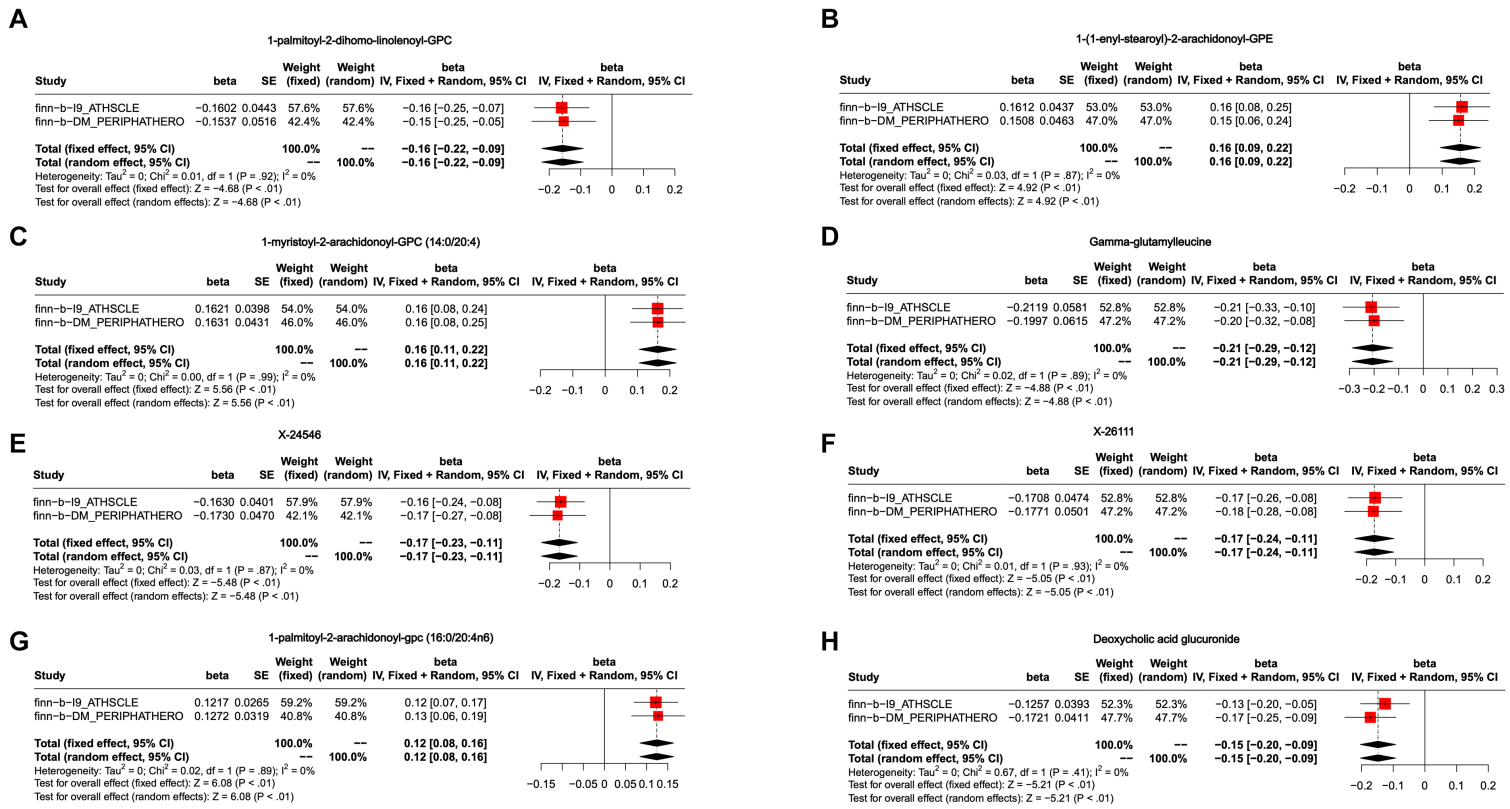
Meta-analysis of the causal associations between blood metabolites and peripheral arteriosclerosis. **(A)** 1-palmitoyl-2-dihomo-linolenoyl-GPC; **(B)** 1-(1-enyl-stearoyl)-2-arachidonoyl-GPE; **(C)** 1-myristoyl-2-arachidonoyl-GPC (14:0/20:4); **(D)** Gamma-glutamylleucine; **(E)** X-24546; **(F)** X-26111; **(G)** 1-palmitoyl-2-arachidonoyl-gpc (16:0/20:4n6); **(H)** Deoxycholic acid glucuronide.

To further enhance the persuasiveness of the estimation, another GWAS data was used for repeated analysis, which registration number finn-b-DM_PERIPHATHERO. There were 206 metabolites significantly associated with peripheral arteriosclerosis (Figure 2B). Through the same sensitivity analysis, there were 21 candidate metabolites that met the criteria (Table 2; Supplementary Figure S1). Combined analysis of the two GWAS datasets, we identified eight candidate metabolites associated with peripheral arteriosclerosis (Figure 3B). Through meta-analysis, we further identified eight candidate blood metabolites (including six known and two unknown) that could influence peripheral arteriosclerosis. In detail, an increase of genetic predisposition of 1-myristoyl-2-arachidonoyl-GPC (14:0/20:4) (OR = 0.16, 95% CI = 0.11–0.22, *p* < 0.01), 1-palmitoyl-2-arachidonoyl-gpc (16:0/20:4n6) (OR = 0.12, 95% CI = 0.08–0.16, *p* < 0.01) and 1-(1-enyl-stearoyl)-2-arachidonoyl-GPE (OR = 0.16, 95% CI = 0.09–0.22, *p* < 0.05) would lead to an increased risk of peripheral arteriosclerosis, while gene susceptibility for higher levels of 1-palmitoyl-2-dihomo-linolenoyl-GPC (OR = −0.16, 95% CI = −0.22– −0.09, *p* < 0.01), X-24546 (OR = −0.17, 95% CI = −0.23- -0.11, *p* < 0.01), Gamma-glutamylleucine (OR = −0.22, 95% CI = −0.29- -0.12, *p* < 0.01), X-26111 (OR = −0.17, 95% CI = - 0.24- -0.11, *p* < 0.01) and Deoxycholic acid glucuronide (OR = −0.15, 95% CI = −0.20- -0.09, *p* < 0.01) predicted a lower risk of peripheral arteriosclerosis (Figure 4).

**Table 2:** MR from blood metabolites on finn-b-DM_PERIPHATHERO.

ID	Metabolites	Method	nSNP	se	*p* value	or	or_lci95	or_uci95
GCST90199741	Gamma-glutamylglycine	MR Egger	26	0.061912	0.025899	0.863271	0.764622	0.974648
		Weighted median	26	0.042659	0.001527	0.87353	0.803462	0.949709
		Inverse variance weighted	26	0.039583	0.002755	0.888245	0.821938	0.959902
		Simple mode	26	0.121099	0.337382	0.888296	0.70061	1.126261
		Weighted mode	26	0.042355	0.003804	0.873599	0.804005	0.949218
GCST90199791	1-arachidonoyl-GPE (20:4n6)	MR Egger	32	0.075342	0.07031	1.151882	0.993747	1.335182
		Weighted median	32	0.051352	0.000239	1.207651	1.092018	1.335528
		Inverse variance weighted	32	0.04173	0.001191	1.144818	1.054909	1.242389
		Simple mode	32	0.135168	0.174129	1.206856	0.925973	1.572941
		Weighted mode	32	0.052248	0.000483	1.225955	1.106624	1.358155
GCST90199849	21-hydroxypregnenolone disulfate	MR Egger	36	0.097588	0.039496	0.811418	0.670156	0.982456
		Weighted median	36	0.061542	0.079813	0.8978	0.795781	1.012897
		Inverse variance weighted	36	0.042671	0.001277	0.871578	0.801648	0.947608
		Simple mode	36	0.125915	0.308245	0.877927	0.685926	1.123672
		Weighted mode	36	0.084599	0.088578	0.862262	0.730511	1.017775
GCST90199867	Tridecenedioate (C13:1-DC)	MR Egger	24	0.102128	0.026949	1.273913	1.042814	1.556225
		Weighted median	24	0.073207	0.053384	1.15191	0.997938	1.329639
		Inverse variance weighted	24	0.050196	0.002711	1.162447	1.053525	1.282629
		Simple mode	24	0.132613	0.156583	1.214359	0.936407	1.574814
		Weighted mode	24	0.108227	0.20475	1.151714	0.93158	1.423866
GCST90199870	4-cholesten-3-one	MR Egger	24	0.087978	0.808403	0.97864	0.823635	1.162816
		Weighted median	24	0.06103	0.474662	1.044597	0.926828	1.17733
		Inverse variance weighted	24	0.043777	0.598731	1.023304	0.939163	1.114983
		Simple mode	24	0.097407	0.828562	1.021564	0.844016	1.23646
		Weighted mode	24	0.072905	0.494944	1.051859	0.9118	1.213434
GCST90199892	Isoursodeoxycholate	MR Egger	20	0.136379	0.186533	1.205971	0.9231	1.575524
		Weighted median	20	0.085258	0.440165	1.068025	0.903668	1.262276
		Inverse variance weighted	20	0.073347	0.540889	1.045871	0.905823	1.20757
		Simple mode	20	0.157214	0.617739	1.083032	0.795826	1.473886
		Weighted mode	20	0.130284	0.696364	1.052975	0.815677	1.359309
GCST90199915	Imidazole propionate	MR Egger	24	0.120278	0.757259	1.03836	0.820286	1.314408
		Weighted median	24	0.080372	0.615218	1.041225	0.889469	1.218874
		Inverse variance weighted	24	0.054116	0.69192	1.021675	0.918858	1.135996
		Simple mode	24	0.128126	0.380456	1.121397	0.872361	1.441525
		Weighted mode	24	0.104805	0.57071	1.062143	0.86491	1.304353
GCST90199937	2-aminooctanoate	MR Egger	27	0.074464	0.257283	0.917294	0.792726	1.061436
		Weighted median	27	0.047971	0.130489	0.930028	0.846569	1.021715
		Inverse variance weighted	27	0.045589	0.79746	0.988369	0.903885	1.080748
		Simple mode	27	0.129477	0.359225	1.128453	0.875529	1.454441
		Weighted mode	27	0.04513	0.037025	0.905552	0.828892	0.989301
GCST90199951	N-acetylalliin	MR Egger	28	0.091904	0.739016	1.031432	0.86141	1.235012
		Weighted median	28	0.059066	0.366769	1.054756	0.939451	1.184214
		Inverse variance weighted	28	0.039318	0.857963	1.007062	0.932368	1.087738
		Simple mode	28	0.124897	0.213878	0.853004	0.667785	1.089596
		Weighted mode	28	0.09317	0.093946	1.175569	0.979356	1.411093
GCST90199958	1-linolenoyl-GPC (18:3)	MR Egger	29	0.165089	0.554655	1.103807	0.798669	1.525526
		Weighted median	29	0.079533	0.541325	1.049779	0.898251	1.226868
		Inverse variance weighted	29	0.066081	0.74087	0.978384	0.859528	1.113676
		Simple mode	29	0.161706	0.422195	1.140789	0.830919	1.566217
		Weighted mode	29	0.154764	0.401932	1.140789	0.842302	1.545051
GCST90199978	Vanillactate	MR Egger	23	0.136027	0.027513	1.380189	1.057182	1.801887
		Weighted median	23	0.085004	0.139481	1.133843	0.959834	1.339398
		Inverse variance weighted	23	0.060122	0.012812	1.161418	1.032313	1.306669
		Simple mode	23	0.161566	0.360481	1.162869	0.847234	1.596094
		Weighted mode	23	0.133007	0.434674	1.111645	0.856542	1.442726
GCST90200326	Gamma-glutamylleucine	MR Egger	16	0.103737	0.016905	0.754893	0.616003	0.925098
		Weighted median	16	0.091276	0.0322	0.822419	0.687698	0.983532
		Inverse variance weighted	16	0.061458	0.001154	0.818944	0.726007	0.923779
		Simple mode	16	0.130633	0.139244	0.815472	0.631266	1.053431
		Weighted mode	16	0.09093	0.044708	0.81941	0.685647	0.979269
GCST90200586	X-21471	MR Egger	25	0.057069	0.001328	0.811859	0.725944	0.907943
		Weighted median	25	0.053056	0.09856	0.916088	0.825609	1.016482
		Inverse variance weighted	25	0.036774	0.000853	0.884579	0.823064	0.950691
		Simple mode	25	0.093757	0.660781	0.959196	0.798179	1.152696
		Weighted mode	25	0.053635	0.039749	0.889914	0.801112	0.988559
GCST90200601	X-23654	MR Egger	38	0.076332	0.06188	1.158474	0.997496	1.345431
		Weighted median	38	0.066793	0.042772	1.144899	1.004413	1.305035
		Inverse variance weighted	38	0.042405	3.79E-05	1.190902	1.095923	1.294112
		Simple mode	38	0.128674	0.990104	1.001608	0.778338	1.288924
		Weighted mode	38	0.107856	0.559433	1.06559	0.862543	1.316435
GCST90200630	X-24546	MR Egger	26	0.080448	0.010369	0.799514	0.682885	0.936062
		Weighted median	26	0.060447	9.45E-05	0.789781	0.701542	0.889119
		Inverse variance weighted	26	0.047032	0.000234	0.841102	0.767034	0.922322
		Simple mode	26	0.119202	0.103207	0.817415	0.647108	1.032546
		Weighted mode	26	0.058348	0.000541	0.793417	0.707677	0.889545
GCST90200670	X-26111	MR Egger	21	0.097531	0.014085	0.768231	0.634559	0.930061
		Weighted median	21	0.06962	0.005744	0.825064	0.719823	0.945691
		Inverse variance weighted	21	0.0501	0.000408	0.837692	0.759343	0.924125
		Simple mode	21	0.1451	0.465963	0.897764	0.675539	1.193092
		Weighted mode	21	0.080028	0.002377	0.757091	0.647183	0.885664
GCST90200685	1-stearoyl-2-arachidonoyl-gpc (18:0/20:4)	MR Egger	36	0.027746	0.000782	1.107777	1.049143	1.169688
		Weighted median	36	0.029979	6.81E-05	1.126821	1.062518	1.195015
		Inverse variance weighted	36	0.022068	6.12E-05	1.092484	1.046238	1.140773
		Simple mode	36	0.075834	0.717878	1.028006	0.886023	1.192742
		Weighted mode	36	0.024484	0.000483	1.098806	1.047322	1.152821
GCST90200692	1-palmitoyl-2-arachidonoyl-gpc (16:0/20:4n6)	MR Egger	31	0.044629	0.1005	1.078654	0.988309	1.177258
		Weighted median	31	0.032818	7.92E-05	1.138292	1.067379	1.213916
		Inverse variance weighted	31	0.031929	6.78E-05	1.135637	1.066747	1.208976
		Simple mode	31	0.107808	0.579151	1.062315	0.859973	1.312265
		Weighted mode	31	0.031911	0.000738	1.127425	1.059071	1.200192
GCST90200699	Deoxycholic acid glucuronide	MR Egger	24	0.078269	0.001759	0.756905	0.649259	0.882399
		Weighted median	24	0.059542	2.92E-05	0.779702	0.693817	0.876217
		Inverse variance weighted	24	0.041132	2.85E-05	0.841857	0.776651	0.912536
		Simple mode	24	0.114368	0.092247	0.81797	0.65371	1.023504
		Weighted mode	24	0.061251	0.000814	0.789889	0.700533	0.890643
GCST90200851	Adenosine 5'-monophosphate (AMP) to glycine ratio	MR Egger	23	0.103826	0.015981	1.312686	1.070982	1.608938
		Weighted median	23	0.068801	0.001127	1.251147	1.093312	1.431768
		Inverse variance weighted	23	0.053484	0.000181	1.221713	1.100128	1.356736
		Simple mode	23	0.132868	0.040154	1.336146	1.029804	1.733618
		Weighted mode	23	0.069214	0.003023	1.259387	1.099621	1.442367
GCST90200988	Benzoate to linoleoyl-arachidonoyl-glycerol (18:2 to 20:4) [1] ratio	MR Egger	15	0.101925	0.006626	0.719775	0.589436	0.878934
		Weighted median	15	0.070918	0.022122	0.850204	0.739871	0.976989
		Inverse variance weighted	15	0.049302	0.002939	0.863616	0.784069	0.951233
		Simple mode	15	0.138835	0.876279	0.978227	0.74518	1.284157
		Weighted mode	15	0.076225	0.003261	0.763471	0.65752	0.886496

### Reverse causation between metabolites and peripheral arteriosclerosis

The reverse causal effect of the peripheral arteriosclerosis phenotype on the identified candidate metabolites was also obtained by MR analysis. In the IVW model, none of the peripheral arteriosclerosis phenotypes had a reverse effect on these candidate blood metabolites at the IV threshold of 5e-08.

### Metabolic pathway analysis

**Figure 5:**
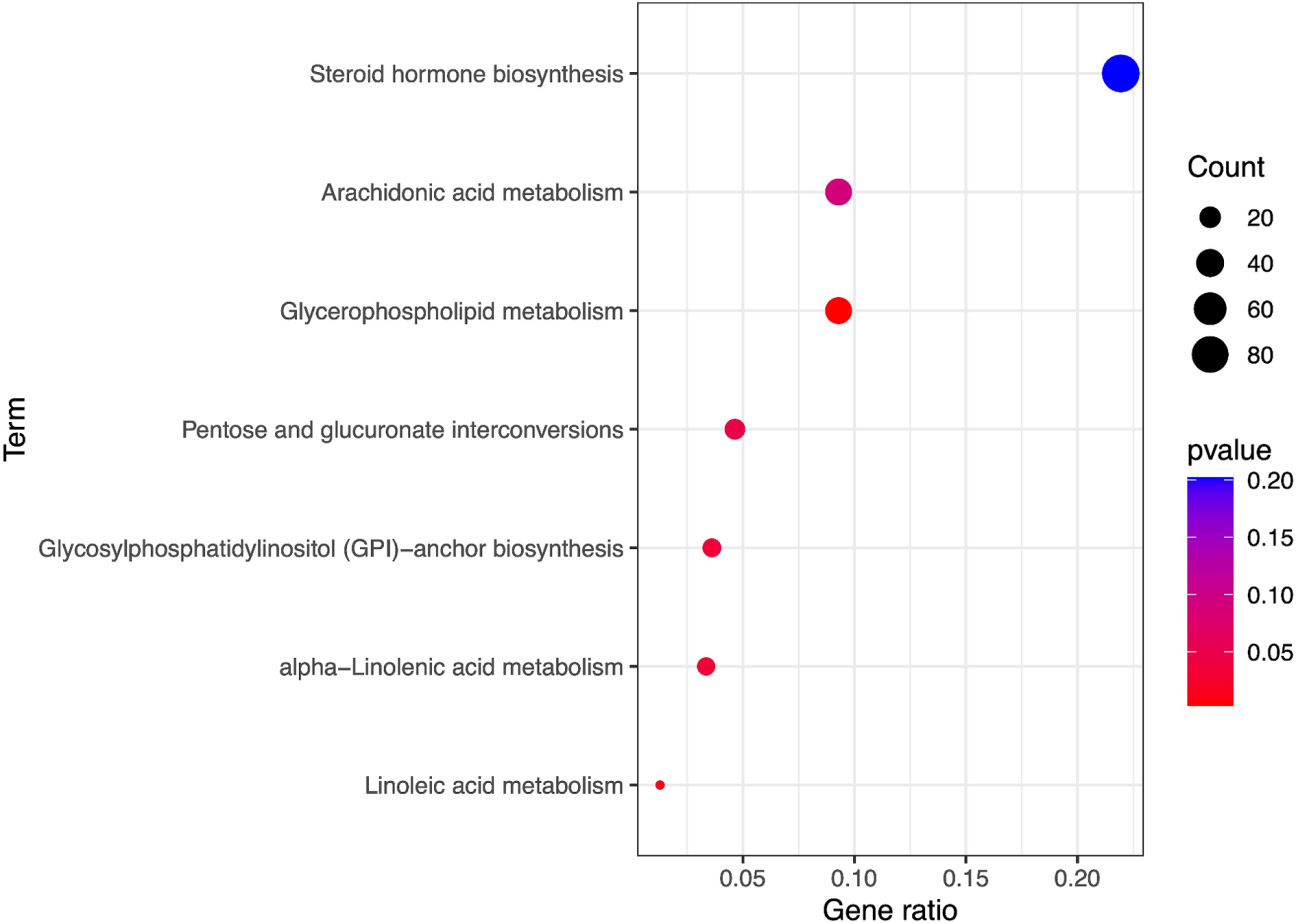
Enriched significant metabolic pathways of eight candidate metabolites.

The metabolic pathway analysis identified five metabolic pathways which may be associated with peripheral arteriosclerosis including Glycerophospholipid metabolism, Linoleic acid metabolism, alpha-Linolenic acid metabolism, Glycosylphosphatidylinositol (GPI)-anchor biosynthesis, Pentose and glucuronate interconversions (*p* < 0.05, Figure 5). The metabolic mechanism of metabolite formation may be involved in the occurrence and development of peripheral arteriosclerosis.

## Discussion

In the present study, the causal relationship between 1,400 blood metabolites and peripheral arteriosclerosis were investigated based on the two large-scale GWAS data and rigorous MR designs. We finally determined that genetically predisposed high levels of 1-myristoyl-2-arachidonoyl-GPC, 1-palmitoyl-2-arachidonoyl-gpc (16:0/20:4n6) and 1-(1-enyl-stearoyl)-2-arachidonoyl-GPE increased risk of peripheral arteriosclerosis, while gene susceptibility for higher levels of 1-palmitoyl-2-dihomo-linolenoyl-GPC, Deoxycholic acid glucuronide, Gamma-glutamylleucine, X-26111 and X-24546 associated with lower risk of peripheral arteriosclerosis. No reverse MR effects of peripheral arteriosclerosis were found on the identified candidate metabolites. In addition, pathway enrichment analysis identified five significant metabolic pathways involved in peripheral arteriosclerosis. As far as we know, this is the first MR study to elucidate the causal relationship between blood metabolites and peripheral arteriosclerosis and incorporate metabolic pathways.

With the aging of the population, the prevalence of peripheral arteriosclerosis will be on the rise (21). Age, high blood pressure, hypercholesterolemia, diabetes, and smoking are recognized risk factors for peripheral arteriosclerosis, but the disease has been developing silently for decades (22). Therefore, one of the major challenges facing clinicians is the early diagnosis of peripheral arteriosclerosis. Although many candidate biomarkers have been proposed, the results have been limited (23, 24). The emergence of new metabolomics techniques provides new ideas for understanding peripheral arteriosclerosis. However, systematic and comprehensive studies on blood metabolomics and peripheral arteriosclerosis are still lacking. Therefore, a critical MR study was conducted to systematically evaluate the causal role of human blood metabolites in the peripheral arteriosclerosis and incorporate metabolic pathways, providing directions for screening and treatment of peripheral arteriosclerosis.

In this study, five blood metabolites were identified to have a protective effect on peripheral arteriosclerosis, including 1-palmitoyl-2-dihomo-linolenoyl-GPC, Gamma-glutamylleucine, Deoxycholic acid glucuronide and two named X- (X-24546, X-26111). However, there are few reports about the related effects of 1-palmitoyl-2-dihomo-linolenoyl-GPC, and the relationship between 1-palmitoyl-2-dihomo-linolenoyl-GPC and peripheral arteriosclerosis deserves further investigation. Glutamylleucine is a dipeptide, a by-product of the synthesis of reduced glutathione catalyzed by gamma-glutamylcysteine synthase (25). Gamma-glutamylleucine has been linked to the risk of cardiometabolic diseases such as obesity, metabolic syndrome, and type 2 diabetes. Meanwhile, the elevated levels of Gamma-glutamylleucine are associated with increased mortality of prostate cancer and play a role in the development of liver-related diseases (26, 27). Gamma-glutamyl dipeptides are involved in a variety of biological activities by activating calcium-sensitive receptors in different organs, including inflammatory activity, oxidative stress, and glucose metabolism. Calcium-sensitive receptors act as G protein-coupled receptors that regulate a variety of cellular processes related to cardiovascular health such as regulating insulin secretion, up-regulating apoptosis and releasing nitric oxide (26). Thus, Gamma-glutamylleucine may have a protective effect in peripheral arteriosclerosis, and its specific biological mechanism remains to be supplemented.

Deoxycholic acid glucuronide binds to the host and participates in the secondary bile acid cycle. Secondary bile acids play an immunomodulatory role by directly affecting the proliferation of immune cells or the production of secondary cytokines (28). Current studies have shown that secondary bile acids are metabolites of gut microbes and are related to cardiovascular health (29). In Diener et al.’s study, Deoxycholic acid glucuronide was shown to be closely associated with the flora, and bile acids can directly drive changes in the gut microbiome (30). This suggests that Deoxycholic acid glucuronide may play a role in peripheral arteriosclerosis by influencing gut microbiome. Recent studies also have found that Deoxycholic acid glucuronide, as a carbohydrate metabolite, is related to the treatment of peripheral artery disease. As a selective serotonin 5-HT2A receptor antagonist, sarpogrelate is excreted mainly in the form of glucuronide conjugate and can be used in the treatment of patients with peripheral artery disease (31). This suggested that Deoxycholic acid glucuronide has great potential in the prevention and treatment of peripheral arteriosclerotic diseases.

We also found that a genetic predisposition to higher levels of 1-myristoyl-2-arachidonoyl-GPC, 1-palmitoyl-2-arachidonoyl-gpc (16:0/20:4n6) and 1-(1-enyl-stearoyl)-2-arachidonoyl-GPE was detrimental to peripheral arteriosclerosis. To date, no association between 1-myristoyl-2-arachidonoyl-GPC and peripheral arteriosclerosis has been reported, with only one study showing that the plasma metabolite 1-myristoyl-2-arachidonoyl-GPC from the microbiota is associated with retained residual beta cell function (32). There are no studies on the relationship between 1-palmitoyl-2-arachidonoyl-gpc (16:0/20:4n6) and peripheral arteriosclerosis. For 1-(1-enyl-stearoyl)-2-arachidonoyl-GPE, Rebholz et al. showed that 1-(1-enyl-stearoyl)-2-arachidonoyl-GPE was significantly correlated with dietary protein intake in patients with chronic kidney disease, which was involved in the metabolism of glycerophospholipid (33). Glycerophospholipid metabolism is the main pathway involved in general systemic immunity and low inflammatory state, indicating that phospholipids are potential inflammatory mediators (34).

In this study, five metabolic pathways were identified that have a causal relationship with peripheral arteriosclerosis, including Glycerophospholipid metabolism, Linoleic acid metabolism, alpha-Linolenic acid metabolism, Glycosylphosphatidylinositol (GPI)-anchor biosynthesis, Pentose and glucuronate interconversions. Glycerophospholipid metabolism has been shown to play a key role in arteriosclerosis (34). Markers of lipid peroxidation damage have been shown to be elevated in patients with cardiovascular disease risk factors. Linoleic acid (LA) is a major dietary omega-6 unsaturated fatty acid, and its metabolite is arachidonic acid (AA). In 30 prospective observational studies in 13 countries, Matti et al. (35) found that the high LA levels were associated with an overall reduced risk of cardiovascular disease, cardiovascular mortality, and ischemic attacks. In randomized controlled feeding trials, dietary polyunsaturated fatty acids (primarily LA) substitutions for carbohydrates or saturated fats reduced low density lipoprotein (LDL) - cholesterol, triglycerides, and ApoB levels, and increased high density lipoprotein (HDL) - cholesterol (36, 37). High blood cholesterol concentration is the main cause of atherosclerosis, and linoleic acid can esterify cholesterol and play a role in reducing serum, liver and blood cholesterol in the body. Meanwhile, conjugated linoleic acid (CLA) is an isomer of linoleic acid. There is evidence that CLA can significantly alter HDL metabolism *in vivo* and mediate the protective effect of atherosclerosis (38, 39). Pentose and glucuronate interconversions are also disrupted in patients with acute coronary syndromes with anxiety disorders (40).

This study has several advantages. First, the present study covered a large scale of genetic variables to analyze the relationship between blood metabolites and peripheral atherosclerosis. Specifically, 1,400 metabolites were covered. Secondly, another set of GWAS data was used for repeated and isolated analysis, which increased the reliability of the results. Third, reverse MR is adopted to avoid reverse causality. Therefore, the causal relationship between metabolites and peripheral arteriosclerosis in this study is considered reliable.

There are still some limitations in the current study. First, metabolomics profiling was conducted using non-fasting plasma samples. Although the metabolomics measurements were adjusted by accounting for the number of hours since last meal or drink, some additional variability may not have been fully captured. Second, the metabolite data are mainly from individuals of European ancestry, and the generality of our findings to other populations deserves further exploration and verification. Third, the efficacy of IV in MR analysis is heavily reliant on the sample size of GWAS. Therefore, larger datasets are needed to enhance the accuracy of the peripheral arteriosclerosis estimates. Fourth, the accuracy of MR analysis depends on the correct interpretation of exposure by instrumental variables, highlighting the need for larger sample sizes to more precisely assess the genetic influence on metabolites. Finally, although this study identified several metabolites associated with the risk of peripheral arteriosclerosis, further research and experimental validation are necessary to confirm these findings.

## Conclusion

In summary, this MR Study identified eight blood metabolites that have a causal effect on peripheral atherosclerosis and identified five metabolic pathways that may be involved in the development of peripheral atherosclerosis. The discovery of these metabolites could be considered as biomarkers for screening and prevention of peripheral arteriosclerosis in the future. The novel contributions of this manuscript include, but are not limited to, our study comprehensively exploring the causal relationship between metabolites and peripheral arteriosclerosis using metabolomic and GWAS data, providing further directions in the development of indicators and a clinical diagnostic panel to aid in the screening of patients who require intervention. We also plan to build a prospective cohort of peripheral arteriosclerosis patients and collect serum samples to clarify the clinical utility of identified metabolites in prediction or diagnosis.

## Data Availability

The data presented in the study were obtained from IEU open GWAS project (https://gwas.mrcieu.ac.uk/) which registration number were the finn-b-I9_ATHSCLE and finn-b- DM_PERIPHATHERO.

## Footnotes

^1^ https://www.ebi.ac.uk/gwas/publications/

^2^ https://gwas.mrcieu.ac.uk/

^3^ https://www.metaboanalyst.ca/

## Supplementary material

Supplementary Figure S1 Sensitivity analysis for candidate blood metabolites on peripheral atherosclerosis GWAS databases of finn-b-DM_PERIPHATHER.

Supplementary Table S1 MR-STROBE checklist.

Supplementary Table S2 The detailed names of 1400 blood metabolite.
